# Integrative Modelling of Gene Expression and Digital Phenotypes to Describe Senescence in Wheat

**DOI:** 10.3390/genes12060909

**Published:** 2021-06-11

**Authors:** Anyela Valentina Camargo Rodriguez

**Affiliations:** The John Bingham Laboratory, NIAB, 93 Lawrence Weaver Road, Cambridge CB3 0LE, UK; Anyela.CamargoRodriguez@niab.com; Tel.: +44-(0)1223-342200

**Keywords:** computational modelling, phenomics, transcriptomics, *Triticum aestivum*, climate resilience

## Abstract

Senescence is the final stage of leaf development and is critical for plants’ fitness as nutrient relocation from leaves to reproductive organs takes place. Although senescence is key in nutrient relocation and yield determination in cereal grain production, there is limited understanding of the genetic and molecular mechanisms that control it in major staple crops such as wheat. Senescence is a highly orchestrated continuum of interacting pathways throughout the lifecycle of a plant. Levels of gene expression, morphogenesis, and phenotypic development all play key roles. Yet, most studies focus on a short window immediately after anthesis. This approach clearly leaves out key components controlling the activation, development, and modulation of the senescence pathway before anthesis, as well as during the later developmental stages, during which grain development continues. Here, a computational multiscale modelling approach integrates multi-omics developmental data to attempt to simulate senescence at the molecular and plant level. To recreate the senescence process in wheat, core principles were borrowed from Arabidopsis Thaliana, a more widely researched plant model. The resulted model describes temporal gene regulatory networks and their effect on plant morphology leading to senescence. Digital phenotypes generated from images using a phenomics platform were used to capture the dynamics of plant development. This work provides the basis for the application of computational modelling to advance understanding of the complex biological trait senescence. This supports the development of a predictive framework enabling its prediction in changing or extreme environmental conditions, with a view to targeted selection for optimal lifecycle duration for improving resilience to climate change.

## 1. Introduction

Senescence, the final, degenerative stage in plant development, occurs in a temporally coordinated manner [1]. The process is triggered by the collective and coordinated assessment of multiple internal and external signals through intricate regulatory pathways [2] and entails many morphological, cytological, physiological, and molecular changes. The first visible sign of leaf senescence is leaf colour degradation, typically from green to pale green to yellow and brown. These changes are caused by rapid chlorophyll degradation during chloroplast degeneration [3] as photosynthesis declines and mesophyll cells lose their ability to produce carbohydrates. Nuclei and mitochondria remain intact until later stages, allowing expression of senescence-associated genes (SAGs) and continued energy production while chloroplasts and their content are already being degraded [4]. These processes are activated to enable the realisation of one of the most important purposes of senescence, namely, nutrient remobilisation, which functions to withdraw and translocate nutrients such as carbon, nitrogen, and other minerals from senescing tissues to other parts of the plant [5]. In annual crops, these prioritised structures are typically fruits and seeds, i.e., those that make up harvestable crop yield. As leaf senescence is accompanied by genome-wide changes in gene expression, the dynamic activation of transcription factors is thought to be a key mechanism that controls the age-dependent expression of the thousands of SAGs. Two transcription factor families, NAC and WRKY, are the major transcription factors that regulate leaf senescence [6]. These transcription factors are also induced by various stress responses, which is consistent with the notion that senescence is an integrated response of plants to both endogenous developmental as well as environmental signals.

Other transcription factors families have been reported to be involved in the regulation of leaf senescence in Arabidopsis thaliana. For example, the related to ABI3/VP1 (RAV) transcription factor family member RAV1 [7] and the R-type MYB-like transcription factor MYBL [8] have both been shown to be positive regulators of leaf senescence. The C-repeat/dehydration responsive element binding factor 2 (CBF2) appears to be a negative regulator of leaf senescence, as its overexpression delays leaf senescence [9]. Several transcription factors that are associated with hormone signalling have also been reported to regulate leaf senescence. For example, Auxin response factor 2 (ARF2) has an important role in modulating auxin-mediated leaf senescence [10], and Signal responsive 1 (SR1), a calmodulin-binding transcription factor, regulates ethylene-induced senescence by directly binding to the EIN3 promoter, a positive transcription factor in the ethylene signalling pathway [11].

As senescence is a key decision point in a plant’s life cycle, delaying leaf senescence (denoted stay green) provides an opportunity to prolong photosynthetic capacity, potentially increasing crop yield [12]. In contrast, premature leaf senescence results in limitations to assimilate production and corresponding yield loss. Premature leaf senescence is triggered by various external factors such as drought [13], salt stress [14], and shading [15], as well as physiological factors such as sugar accumulation content [16] or plant hormone levels [17,18]. Therefore, the onset of senescence is an important adaptive feature with individual plants integrating factors in the environment with internal signals to trigger senescence via regulatory networks. On the evolutionary level, these mechanisms themselves are subject to selection.

Therefore, an in-depth understanding of the molecular mechanism of senescence is important in delaying leaf senescence and increasing cereal crop production. The genetic information encoded in an organism’s DNA is transferred into a functional gene product via the process of gene expression, which leads to the formation of a phenotype [19]. The formation of such phenotypes is usually governed by multiple temporal and spatial factors [20], and their variation can be attributed to the collective response of multiple small effects associated with the phenotype. These changes can be due to phenotype-associated genes that turn on or off at various times. In other words, phenotype formation is, in part, governed by genes whose effects change with time and is commonly the result of the interaction of multiple genes that together make up gene regulatory networks (GRN).

Studying the dynamics of gene–gene interaction over time is crucial to understanding the properties and functions of genes, which helps reveal the genetic architecture of complex traits and other biological functions. GRN’s inference can be done via in vitro genetic experiments, such as comparative screening of mutants vs. wild types for a trait or gene of interest. As mutations can interrupt cellular processes, specific genetic or phenotypic mutants are often used to understand gene function and interaction. This approach can be costly and time-consuming and is difficult to scale for complex traits, as it would require reverse engineering of single and combined mutants from the target organism. An alternative is to infer GRNs based on measurement of gene expression to reveal gene expression patterns affected by single or multiple factors such as time or environment. Many methods have been proposed to infer GRNs from gene expression data, including Bayesian networks [21], which are graph-based models of joint multivariate probability distributions that capture properties of conditional independence between a set of genes; Boolean networks [22], where a set of Boolean variables and Boolean functions are used to describe gene–gene interactions; ordinary differential equations [23], which use a set of differential equations to directly describe dynamic changes of the mRNA content in a precise manner; random forest [24], which builds multiple decision trees to infer the gene–gene interaction; and regression type methods [25], which use standard regression and model shrinkage techniques to select parsimonious, predictive models for the expression of a gene or cluster of genes as a function of gene expression, environmental influences, and time.

Expression regulation is dynamic, and time-course data can be used to infer causality, but available gene expression datasets tend to be short or sparsely sampled. In addition, temporal methods typically assume that the expression of a gene at a time point depends on the expression of other genes at only the immediately preceding time point, while other methods include additional time points without any constraints to account for their temporal distance. These limitations can contribute to inaccurate networks with many missing and anomalous links.

The molecular processes involved in leaf senescence are known to involve time-dependent interactions with both internal (developmental) and external (environmental) signals [25]. This requires a highly integrative process towards cell death with nutrient recycling and storage [26]. In this study, we use a multiscale modelling approach to integrate transcriptomic and digital time series phenotypic data to simulate the senescence process in wheat. While multiscale modelling is far from popular and multiscale modelling of senescence has never been developed, other biological processes have been attempted, mainly in the Arabidopsis Thaliana plant model, using this strategy. For example, root gravitropism [27] was simulated by integrating phenotypic data from a DII-VENUS essay in conjunction with a mathematical model to quantify auxin redistribution following a gravity stimulus. Another study used a mathematical model of a small GRN to demonstrate how two nested feed-forward loops precisely control asymmetric cell divisions within the root stem cell niche [28]. One of the most complete multiscale plant models to date is a family of Arabidopsis framework models [29,30], which link genetic regulation and biochemical dynamics to organ and plant growth. The model showed that increasing leaf production rate in developmentally misregulated transgenic Arabidopsis sufficiently explained the smaller leaf size phenotype of this transgenic. The model was also used to predict phenotypic responses due to altered circadian timing in clock-mutant plants [30]. More advanced multiscale models have been used to explore the impacts of genetic modifications to soybean photosynthesis in ambient and elevated carbon dioxide [31] and to explore potential gene engineering strategies for producing trees with improved bioenergy traits while mitigating negative impacts on tree growth [32].

Wheat is a major cereal crop, and the ability to predict and/or manipulate senescence offers the opportunity to delay leaf senescence to increase harvestable grain yield [33]. The developed model describes temporal gene regulatory networks and their effect on plant morphology leading to senescence. The use of digital plant development data extracted from images captured using a phenomics platform adds important resolution to the description of plant development. The modelling assessment approach described here is designed to facilitate the integration of a broad range of phenomics and transcriptomics data and facilitates future expansion to include environmental measurements (such as temperature and light conditions). This study demonstrates the potential of applying computational modelling to predict a complex, dynamic phenotype based on a novel approach to integrate transcriptomic and phenomics data. 

## 2. Results

### 2.1. Gene Expression Profiles Vary across Genes and Time Points

**Figure 1:**
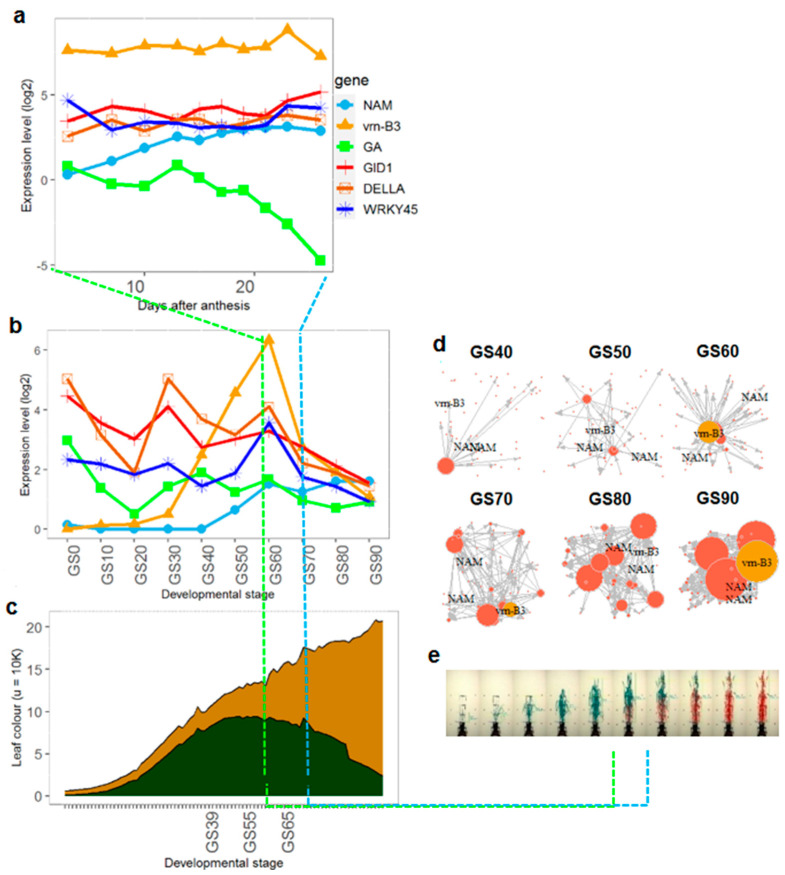
Moving beyond the limits of current senescence research: an integrative approach towards dissecting senescence. Gene expression profiles, from leaf tissues, corresponding to members of the NAM gene family (light blue) and VRN-B3 (amber) at (**a**) a narrow developmental window around anthesis (the traditional approach), contrasted with (**b**) our analysis across the wheat lifecycle, from seedling emergency to grain ripening (see Table 1 for growth stages), using public data. (**c**) Colour plot describing average colour changes of MAGIC lines over the lifecycle. Green and senesced areas in the plant are represented by green and yellow, respectively (see Table 1 for growth stages). (**d**) Lines and parents of the wheat MAGIC population imaged across their lifecycle. (**e**) Topological senescence gene networks from GS40 to GS90 inferred from (**b**).

Expression of senescence-associated genes in Chen’s model [34] show varying profiles post-anthesis (from 3 to 26 days after anthesis (GS60)) depending on gene type (Figure 1a). vrn-B3 expression is highest overall, showing consistent patterns of expression post-anthesis. Across the lifecycle, vrn-B3 expression increases rapidly at GS30 to a peak at GS60, which corresponds with flowering time, indicating that leaf senescence in wheat is partially dependent on phenology, as demonstrated in Arabidopsis and barley [16] reaching maximum expression at anthesis. 

Previous studies have suggested that NAM-B1, B2, A1, D1, and D2 might be redundant [35], and we found high correlation (>0.9) between NAM gene expression profiles. The NAM genes start with low gene expression (<0.5 log2), increasing at around GS50. Expression patterns across time from the seedling to ripening stage also showed significant variation depending on gene type (Figure 1b). This shows a rapid increase in the NAM gene post-anthesis, which reaches a plateau at around 15 dpa, consistent with the results presented in Figure 1a.

Phytohormones Gibberellins (GA) have been reported to promote senescence via complex interconnecting pathways and cytokinins and auxin with senescence inhibition [34]. GA serves as an important endogenous signal to regulate age-dependent leaf senescence. GA’s expression is downregulated during and after anthesis in Figure 1a, and variable in Figure 1b. The expression profiles for GID1, DELLA, and WRK45 follow similar patterns post-anthesis (Figure 1a) and throughout the lifecycle (Figure 1b), suggesting involvement in similar pathways. Previous work has shown that WRKY45 may positively regulate age-dependent leaf senescence via the GA pathway [34]. 

### 2.2. Colour Distribution

Average changes in plant colour across time and their relation with wheat development are shown in Figure 1c. The plot shows that leaf senescence, represented by yellow, increases at a faster rate after GS39 when the flag leaf has fully emerged. At GS65, plant vegetative areas are senescing at a much faster rate. Both Figure 1a,b show significant changes between GS40 and GS50 growth stages.

When comparing Figure 1a,b NAMs and Vrn-B3 genes show an increase in expression at the same time as plant greenness starts a rapid decline towards senescence (indicated by the decrease in green pixels in the graph). However, while NAMs remain up-regulated after anthesis, Vrn-B3′s expression seems to show a declining pattern after the same developmental stage.

### 2.3. GRN Reconstruction Reveals Interactions across Developmental Time Points

Filtering for significantly differential expression (SDE) resulted in 12944 genes of which only two NAM genes (NAM_A1 and NAM_D1) and the Vrn-B3 gene were SDE. The remainder of the genes present in Chen’s model were not SDE and were therefore filtered out.

In order to focus on senescence-related genes, all the genes in the same clusters of NAM-A1 and NAM-D1 were used in the GRN de novo reconstruction, across 10 wheat developmental stages (GS10 to GS90). When running the simulation, network reconstruction was not possible before GS30, as there were fewer recurrent interactions up to that time point. Therefore, GRN reconstruction goes from GS40 to GS90 (Figure 1e). Briefly, at GS40 (A) the network has a single hub (node size dictated by number of interactions), TraesCS6D02G082300.1, and the rest of the genes are not connected. At GS50, the network has two main hubs (TraesCS3A02G092000.1 and TraesCS3D02G024700.1), and the remaining genes are unconnected. At GS60, the network has two super-hubs (link hubs), one is TraesCS4A02G485000.2 and the other is TraesCS7B02G013100.1 (Vrn-B3), and the latter interacts with the genes NAM-A1 and NAM-D1. From GS70 to GS90, the network has more super-hubs with vrn-B3 remaining, but no connecting edges between vrn-B3 and NAM-A1 and NAM-D1.

### 2.4. In Silico Reconstruction of GRN Modelling Senescence

**Figure 2:**
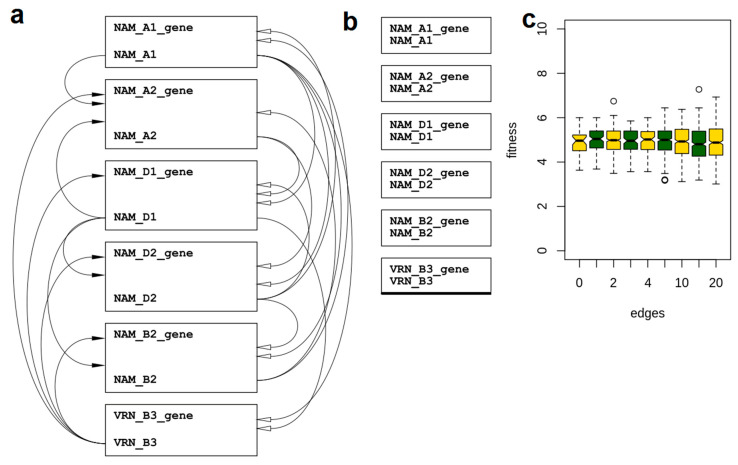
In silico simulation of GRN using a variable number of interactions (edges between genes), which were rewired at random. (**a**) GRN with 20 edges, where black arrows indicate activation, white arrows indicate repression; (**b**) GRN 0 edges; and (**c**) correlation between in silico and observed data.

As genes Vrn-B3, NAM-A1, and NAM-D1 showed a possible interaction at anthesis (GS60), all the NAM genes (NAM-A2, NAM-B2, NAM-D2, NAM-A1, and NAM-D1) together with Vrn-B3 were used to in silico test the hypothesis of the Vrn-B3 and NAMs interaction towards the onset of senescence. The transsys representation of a possible interaction between these genes is shown in Figure 2a. To identify the most likely gene-interaction model that could reproduce the empirical data, networks with six nodes (i.e., genes) and 1, 2, 3, 4, 5, 10, 15, and 20 edges E (E = 1, 2, 3, 4, 5, 10, 15, and 20) were generated. For each E topology, 20 samples were generated, each representing the random rewiring of a given E number of edges. A null model (Figure 2b), with no edges between nodes, was added to the search set. The numeric values in the transsys programs were initialised with random numbers drawn for a uniform distribution and subsequently optimised (20 runs) in order to achieve a close fit to the empirical data. Results of optimisation shown in Figure 2c suggest that an interaction between the NAMs and Vrn-B3 was not likely, as the null model scored similarly to the other models where at least one interaction was modelled. In addition, fitness (e.g., correlation metric between empirical and synthetic data) between empirical and synthetic data was worse when more edges were added to the model.

### 2.5. Modelling the Senescence Phenotype

**Figure 3:**
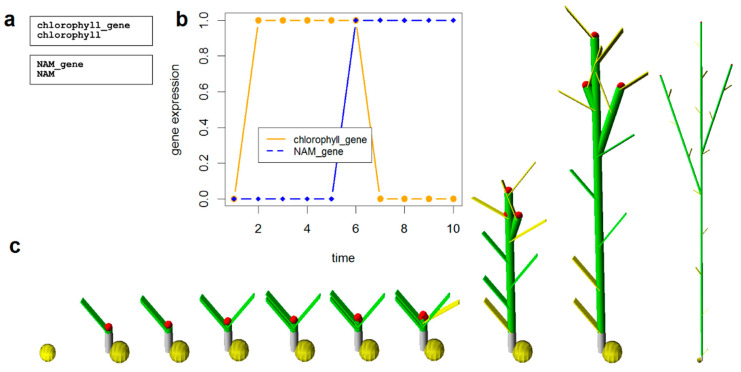
Modelling the senescence phenotype using an alternative strategy with (**a**) two hypothetical genes, (**b**) when the NAM gene is upregulated, the chlorophyll is downregulated, and therefore senescence is triggered, which is also graphically modelled in (**c**), where the model plant starts to become yellow at step number 7 (or 6 beginning from emergence).

The last task of this study was to simulate the senescence phenotype based on gene regulation. Since no interactions between the NAMs and the Vrn-B3 genes were founded at the in silico reconstructing the GRN modelling senescence step, an alternative strategy was used to be able to simulate the senescence phenotype as a function of time. The alternative strategy was based on the assumption that plant senescence and therefore chlorophyll degradation is triggered by a cascade of signals that under ideal circumstances are influenced by plant age. Figure 3a is a network with two genes (and products), one representing signal cascades (NAM_gene) and the other representing chlorophyll degradation (chlorophyll_gene). Figure 3b shows gene expression of these two genes across time, when NAM_gene is upregulated, the hypothetic chlorophyll_gene becomes downregulated, and therefore senescence is triggered, which is demonstrated graphically in Figure 3c. Time one in the plot and in the plant model represents emergence, and time seven represents anthesis (GS60). This simple model is able to describe in a very simple manner how senescence is triggered at the molecular level and how that can be represented using an in silico model of a plant.

## 3. Discussion

In this study, a data-driven approach to understanding the biological process of senescence in wheat is reported. Senescence is a highly synchronised process that involves the cross-talk between genes, or groups of genes, at given time points during plant development. The observed senescence phenotype is the combination of multiple pathways that are constantly sensing internal and external cues to trigger the underlying processes.

The study of senescence in plants has mainly been explored in plant models such as Arabidopsis. These studies have reported genes associated with the senescence phenotype, predominantly based on gene function, and their expression patterns pre- and post-leaf decolouration. Such senescence-associated genes (SAG) usually belong to the NAC, WRKY, and MYB families. The rapid development of new omics technologies has made plant screening at the molecular and organ level faster and cheaper, allowing extension of work from models into crop species such as wheat [36]. The recently sequenced and annotated RefSeq v1.0 [37] wheat genome and the currently available wheat TILLING population [38] now allow for detailed functional characterisation. Data generated from this study were used to identify the NAM transcription factors NAM-A2, NAM-B2, NAM-D2, NAM-A1, and NAM-D1 as likely involved in the triggering of senescence in wheat [12].

Pathway reconstruction in wheat is now also possible given the availability and consolidation of high-quality gene expression data produced by more than 100 wheat experiments [39]. These data, plus ongoing annotation of multiple wheat genomes [36], offer new opportunities to use mechanistic and machine learning methods to understand the senescence process in wheat and to develop in silico models to inform lab-based research.

This study collected and mined heterogeneous data from several publicly available wheat experiments and linked them with currently available models of senescence. Data from previous QTL mapping that were a [40] potential link between the Vrn-B3 flowering time gene and the onset of senescence were also added to the analysis. Previous work has shown a link between flowering time and senescence. Miryeganeh et al., (2018) [41] observed a synchronisation of Arabidopsis flowering time and whole-plant senescence, and Xie et al., (2016) [42] suggested that leaf senescence in wheat is partially dependent on the genetics of the flowering system based on QTL analysis. However, the mechanisms underlying this interaction are still unknown.

Gene expression analysis carried out in this study confirmed that genes NAM-A1 and NAM-D1 were significantly expressed prior to anthesis, as reported by previous studies on senescence in wheat [43]. NAM genes are also associated with increasing wheat grain protein, zinc, and iron content and with the onset of senescence [43,44]. Silencing of NAM genes resulted in decreases of 30%, 36%, and 38% for GPC, iron, and zinc, respectively [44]. Recently, NAM orthologous genes have been identified in *Hordeum vulgare* and *Triticum timopheevii Zhuk*; these have been shown to have the same function [17].

The Vrn-B3 flowering was also significantly expressed prior to anthesis. Other candidate genes analysed in this study such as WRKY45, GID1, and the DELLA were not significantly expressed but showed a similar expression pattern across time. We speculate that perhaps the role of WRKY45 is not specific to senescence, but this can only be tested using mutants.

Although clustering analysis of SGE genes placed Vrn-B3 and the NAM genes within the same cluster, the genes were only linked at anthesis. This result was used to attempt to reconstruct an in silico GRN describing senescence using the Vrn-B3 and the NAM genes as nodes in the network. After testing multiple models, hypotheses were made on how genes candidate interacted; none of the models produced synthetic data that were similar (in correlation terms) to the empirical data. This result suggests that either there is not an interaction between Vrn-B3 and the NAM genes or that key senescence genes that link Vrn-B3 and the NAM are missing.

Since the ultimate task of this study was to model the senescence phenotype based on the GRN reconstruction, but no strong evidence was found regarding the interaction between Vrn-B3 and the NAM, an alternative strategy was used to still be able to model the phenotype. The new strategy used what is widely known about senescence (i.e., a cascade of signals induce chlorophyll degradation and therefore leaf yellowing) to reconstruct a simple model of senescence. This simple model was used as the molecular base to model the senescence phenotype. The resulted model was able to show the initiation of leaf senescence at anthesis.

Future work is required to develop a full in silico model of senescence in wheat. Model reconstruction can now draw on recently available omics resources and use a combination of advanced machine learning methods. This will support the prediction of gene interaction-based predictors such as time, phenotype, gene function, and current gene associations. An accurate in silico model of senescence will inform and add value to functional validation experiments including the screening of mutants under an array of treatment conditions. This will support understanding of gene function and gene–gene interactions for this agronomically important and genetically complex developmental process.

## 4. Materials and Methods

Despite the role that senescence plays in grain filling, in wheat, there is not currently a simple model that attempts to describe the senescence process. This study uses a number of omics datasets as well as a combination of computational methods to attempt to reconstruct a simple model of senescence in wheat. This model is by no means exhaustive, as the idea of the study is to illustrate a data-driven strategy to model key biological processes. A such, a small number of genes are selected as likely to be involved in senescence, and a GRN reconstruction strategy is followed to identify gene–gene regulation.

**Figure 4:**
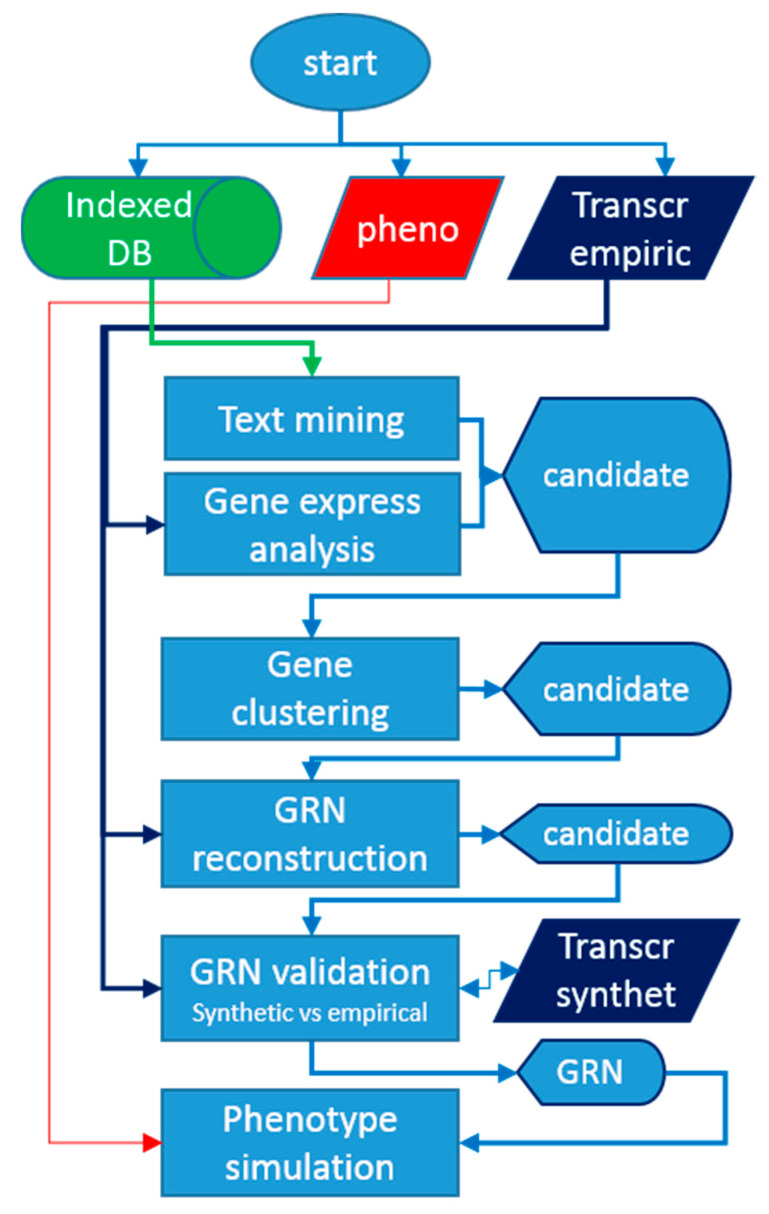
Data integration and modelling pipeline.

The pipeline, depicted in Figure 4, starts from sourcing omics data, followed by candidate identification, which goes from a large list to a trimmed down set, and finally, modelling the senescence at the molecular and plant level. 

### 4.1. Plant Material and Imaging

A subset of the NIAB Elite eight-founder MAGIC wheat (*Triticum aestivum* L.) population described in [40,45,46] was used in this study to analyse trait development over time. Full details of the population including complete pedigrees, genotypes, and existing phenotyping data can be found at https://www.niab.com/research/agricultural-crop-research/resources/niab-magic-population-resources, accessed on 1 May 2020.

As described in [40,45] MAGIC seeds were sown, and emerged seedlings vernalised for nine weeks. Following vernalization, plants were placed on a Smart House conveyor system to allow for regular automated imaging. Daily imaging was controlled by a LemnaTec 3D Scanalyzer (LemnaTec, GmbH, Wuerselen, Germany) for image acquisition. Four RGB pictures (2056 × 2454 pixels) were taken of each plant, one top view and three side views with a 45° horizontal rotation. Once the ears started to ripen, plants were removed from the system and allowed to finish ripening naturally with reduced watering, and manual photographs were taken at final maturity. 

Plants were also manually scored for developmental stages according to the Zadoks scale [47] three times per week to assess the number of days after sowing (DAS) to GS39 (flag leaf fully emerged), GS55 (ear 50% emerged), and GS65 (50% anthesis). 

Digital images were processed using Matlab [48]. Briefly, RGB images were quantized (compressing a range of colour frequencies to a single value) to reduce the number of distinct colours down to 128. Second, frequencies were calculated per colour. A matrix was produced where rows corresponded to images (per MAGIC line per time point), and columns were the colour frequencies. Third, frequencies corresponding to background elements such as tray, cage, background wall, carriage, and labels were filtered out.

### 4.2. Gene Expression Analysis and Gene Selection

We used the Wheat Expression Browser [39], a high-quality annotated reference wheat expression database, to search for expression profiles of senescence-associated (SA) genes. We selected samples extracted from leaves and shoots tissues, across nine wheat developmental stages (Table 1) in the absence of a treatment. This query returned 209 samples and 77,533 transcripts.

expVIP uses standard normalization methods for different RNA-Seq platforms and scaled between experiments to make the expression values of multiple arrays comparable. 

We also looked at gene expression data screened by Borrill et al., (2019) [43] to identify transcription factors associated with senescence. Data were produced from wheat leave tissues collected at 3, 7, 10, 13, 15, 17, 19, 21, 23, and 26 days after anthesis (daa) and also deposited in the Wheat Expression Browser [39]. 

To analyse genes known to be associated with senescence across time, we searched for senescence models already reported in the literature and selected the model with most genes presented in the expression data set. The model chosen, reported in Chens et al., (2017) [34], contained genes from the families DELLA, GA, GID1, WRKY45, and SAGs. Since Chens’ model did not name particular SA genes, we looked for specific SA genes such as the NAM transcription factors NAM-A2, NAM-B2, NAM-D2, NAM-A1, and NAM-D1, because they have been previously shown to contribute to the triggering of senescence in wheat [43,44].

In addition to the genes in Chen’s model, we also included the Vrn-B3 gene because of its likely association with senescence. Xie et al., (2016) [42] screened a wheat mapping population to understand the physiological and genetic relationships among anthesis time, leaf senescence, grain filling processes, and individual grain weight. They identified a QTL for anthesis dates on chromosome 7B, which coincided with the duration and rate of rapid senescence [42]. Camargo et al., (2018) [40] screened a wheat MAGIC population to identify QTL marker association with senescence. They also found a QTL on the same region of the Vrn-B3 gene.

### 4.3. Selection of Senescence Clusters

Gene expression and clustering analysis were performed to identify a subset of potential senescence-associated genes. First, transcripts were filtered if their total number of counts was less than or equal to 10 (≤10). Second, differential expression analysis was performed using the DESeq2 package [49], which uses Negative Binomial distribution to identify gene differential expression. Gene expression was calculated over the time series. Third, candidate transcripts were selected when Padj ≤ 0.05 and log2FoldChange (≥2 and ≤−2). Fourth, Robust Sparse K-means (RSKC) [50] was applied over the set of candidate genes. Robust sparse K-means is a clustering method that extends the sparse K-means clustering method to make it resistant to outliers by trimming a fixed proportion of observations in each iteration. The arguments to run the RSKC function were ncl = 30 (the number of clusters to be identified), which was chosen with the idea to decrease the number of genes per cluster and thus test smaller networks at the GRN reconstruction phase. GRN reconstruction requires testing all possible combinations of genes within a set; testing becomes expensive and sometimes purposeless. α = 0.1 (the proportion of observations to be trimmed). L1 = 5 (the upper bound for the L1 constraint for the vector of weights w). nstart = 300 (the number of random initial sets of cluster centres in every step (a), which performs K-means or trimmed K-means).

These outliers are flagged both in terms of their weighted and unweighted distances to eliminate the effects of outliers in the selection of feature weights and the selection of a partition. Fifth, genes within the same cluster of NAM genes were selected. Sixth, gene expression profiles of candidate genes were used as the empirical data set.

### 4.4. Gene Regulatory Network Inference

In order to avoid the limitations of short or sparsely distributed temporal gene expression data, we used the time-lagged ordered lasso, a regularized regression method with temporal monotonicity constraints, for de novo reconstruction of GRNs [51,52]. The time-lagged ordered lasso is based on the ordinary lasso [53], which performs feature selection and regularization for model fitting and uses an order constraint, in this case, to reflect the relative importance of the features. Specifically, the time-lagged ordered lasso makes the following assumptions for the de novo reconstruction of GRNs: (a) The expression of a gene is linearly dependent on the expression of its regulators at multiple preceding time points. (b) The temporal distance between a target gene variable and a lagged variable of a predictor gene increases whilst the regulatory influence of the lagged variable on the target decreases.

The time-lagged ordered lasso reconstructs a GRN as follows: for each gene *i* in a time-course expression dataset, an expression model is fitted with maximum lag *l_max_* allowed by the data and lasso regularization [53]. Model fitting is performed by solving the following problem using the time-lagged ordered lasso:

(1) $$\underset{\left\{w_{ji,k}\right\}}{\min}\frac{1}{2}\overset{T}{\underset{t=1}{\sum }}{\left(x_{i}\left(t\right)-\overset{p}{\underset{j=1}{\sum }}\overset{l_{max}}{\underset{k=1}{\sum }}w_{ji,k}x_{j}\left(t-k∆t\right)\right)}^{2}+\lambda \overset{p}{\underset{j=1}{\sum }}\overset{l_{max}}{\underset{k=1}{\sum }}\left|w_{ji,k}\right|$$

subject

$$\left|w_{ji,k}\right|\;\geq \left|w_{ji,k}\right|\geq \ldots \;\geq \;\left|w_{ji,k,\;l_{max}}\right|,$$

where $x_{i}\left(t\right)$ is the expression of gene *i* at time *t*, and the monotonicity constrain $\left|w_{ji,k}\right|\geq \cdots \;\geq \;\left|w_{ji,k,\;l_{max}}\right|$ encodes the time-lagged order assumption of the expression model. Thus, an edge from gene *j* to gene I is predicted if any of the coefficients $w_{ji,1},\;\ldots ,\;w_{ji,\;l_{max}}$ of *j*’s lagged variables are not zero.

Results from the time-lagged ordered lasso de novo reconstruction are binary adjacency matrices. The elements of the matrix (i.e., 0, 1) indicate whether pairs of vertices are adjacent (1) or not (0) in the graph. Adjacency matrices were plotted using the R package igraph [54].

### 4.5. GRN Simulation Transsys

**Figure 5:**
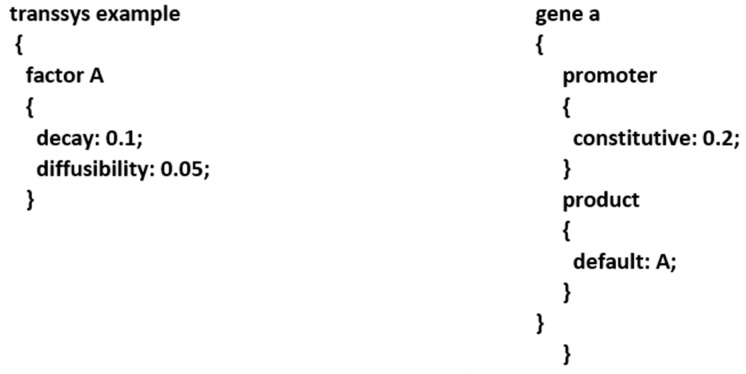
Example transsys programs comprising one factor, A, and one gene, a. More details modelling GRN using transsys are available on http://www.transsys.net, accessed on 1 May 2020.

Transsys [55] is a framework for computational RGN modelling that formally represents RGNs as transsys programs (Figure 5).

A transsys program has two types of components, genes and factors. Factors can be used to model transcription factors as well as any other effector molecules that impinge on gene expression. Each component is specified by an individual declaration. Factor declarations contain the gene product’s decay rate. Gene declarations contain a promoter block and a product block. The promoter block represents the cis-regulatory information of a gene and is composed of promoter elements. It determines the gene’s rate of expression as a function of the expression levels of the regulators of the gene. Each promoter element specifies a rate of expression. Constitutive promoter elements specify a fixed expression rate. Activate and repress elements state a trans-acting factor that activates or represses the gene, respectively. The strength of activation or repression is determined by a Michaelis–Menten function, which is parametrized by vmax, the maximal regulatory effect, and KM, the concentration of the trans-acting factor at which it exerts half its maximal effect. Transsys simulates temporal dynamics of gene expression. Given a transsys program and the current expression levels of factors, a single update is the computation of the expression levels in the next time step. Repeated updates computed in this way generate synthetic expression profiles of the factors. Here, synthetic expression profiles were compared against empirical measurements in order to evaluate transsys programs for their ability to explain empirical data. 

### 4.6. Network Parameter Optimization and Dissection of GRN Topology

The parameters of the target genes and their products, as well as the identification of the best target topology, among a series of candidate models, were inferred using the DoGeNetS (discrimination of gene network structures) method [56], which addresses the research challenge of quantitatively and objectively comparing candidate structural models where most numerical parameters are not determined. DoGeNetS aims to discriminate computational models of GRN structure according to their ability to reproduce a set of gene expression measurements (synthetically or empirically generated data). DoGeNetS requires a target matrix of gene expression data and a set of candidate networks as input. The empirical data set filtered at the selection of senescence clusters was used as the target matrix of gene expression data. The transsys framework was used to represent candidate networks and to compute synthetic gene expression matrices by numerical simulation. Thus, random transsys programs generated using a scale graphs strategy, characterised by a power-law distribution of both incoming and outgoing degrees, were used to generate the topologies. Power-law networks are regarded as a ‘universal’ model, because they possess a number of important properties, such as the presence of hubs and large numbers of nodes with few connections as well as a typical small-world behaviour [57]. This model is characteristic of GRNs. The networks here had N number of nodes (i.e., gene and gene’s products) and E number of edges (i.e., interaction between genes). For each E topology, 50 samples were generated. The numeric values in the transsys programs were initialised with random numbers drawn for a uniform distribution and subsequently optimised in order to achieve a close fit to the empirical data. The SimGenex [58] component of transsys was used to guide the simulation of gene expression time series. Fitness between empirical and synthetic data was measured using Pearson correlation. DoGeNetS scores the divergence of the synthetic matrices from the target matrix, and it optimises the numerical parameters in each candidate network to minimise the difference. These optimised divergence scores are the basis for identifying the candidate topology that is most consistent with the target gene expression matrix. In this simulation, a fitness score equal to six meant that there was no similarity between the empirical and the synthetic data. A score greater than six suggested a negative correlation between empirical and synthetic data. A fitness score equal to zero meant high similarity between the empirical and the synthetic data.

Target networks were integrated into an L-transsys Lindenmayer system. The development of this system was simulated for a fixed time interval, after which expression data on all genes of the target network were collected from the grown structure. For each gene in the target network, a knockout mutant was generated, and gene expression values were collected. The resulting data set was used as input for regulatory network reconstruction. Reconstruction was evaluated by comparing the reconstructed network to the target network.

## Figures and Tables

**Table 1:** Wheat developmental stages.

Stage	Name
GS10	Seedling growth
GS20	Tilling
GS30	Stem elongation
GS40	Booting
GS50	Inflorescence emergence
GS60	Anthesis
GS70	Milk development
GS80	Dough development
GS90	Ripening

## Data Availability

The data used in this study can be found as follows: Transcriptomic data are available on the Wheat Expression Browser [39]. Digital images are available in the Aberystwyth research data repository (https://www.plant-phenomics.ac.uk, accessed on 1 June 2016), corresponding to the W8 experiment.
